# Essential work, unequal places: Neighborhood characteristics of the essential workforce in the United States during the COVID-19 pandemic

**DOI:** 10.1371/journal.pone.0357792

**Published:** 2026-09-30

**Authors:** Kate Duchowny, Vicky Wang, Grace Noppert, Robert Melendez, Lindsay Gypin, Philippa Clarke

**Affiliations:** University of Michigan, Institute for Social Research, Survey Research Center, Social Environment and Health, Ann Arbor, Michigan, United States of America; International Institute of Information Technology Bangalore, INDIA

## Abstract

**Introduction:**

At the onset of the COVID-19 pandemic, more than 55 million U.S. workers continued providing “essential” services crucial to public health, safety, and economic stability, even as large segments of the workforce sheltered at home. Despite the central role these workers played in safeguarding the economy and the greater American public, little is known about the neighborhood environments in which essential workers lived and how well these communities were equipped to support the health and well-being of essential workers during the pandemic. Therefore, the primary objective of this study was to describe the social and physical characteristics of U.S. neighborhoods with varying concentrations of essential workers during the COVID-19 pandemic.

**Methods:**

We linked data from the National Neighborhood Data Archive (NaNDA) with American Community Survey (ACS) 2016–2020 estimates to identify the residential concentration of essential workers across U.S. census tracts. Neighborhoods were categorized into quintiles based on the proportion of essential workers. We examined access to key neighborhood resources relevant to pandemic resilience, including food outlets, health care facilities, and parks/green space. We summarized characteristics using descriptive statistics and heatmaps. Differences between the highest (Q5) and lowest (Q1) quintiles were assessed using Welch’s two-sample t-tests.

**Results:**

Neighborhoods with higher concentrations of essential workers had greater access to grocery stores but significantly less access to health care facilities and parks/green space compared with neighborhoods with fewer essential workers. These disparities highlight uneven community support for those workers required to continue in-person work during a national public health emergency.

**Conclusion:**

Using nationwide neighborhood and census-tract data, we found that neighborhoods with a high proportion of essential workers generally had better access to food stores but substantially lower access to health care and parks/green space. Recognizing these uneven community conditions is critical for strengthening preparedness efforts and ensuring that neighborhoods are better equipped to support the essential workforce during future disasters and public health crises.

## Introduction

In March 2020, ‘stay at home’ orders were enacted throughout the United States due to the COVID-19 pandemic, which fundamentally transformed daily life and highlighted the pivotal role residential neighborhoods play in shaping health and well-being [1]. With the majority of public spaces closed during this time, one’s immediate neighborhood environment became critical to accessing essential resources such as food outlets, health care services, and opportunities for outdoor activity, all of which have been repeatedly shown to influence health promotion and risk mitigation, especially during public health disasters [2,3].

The neighborhood context has long been recognized as a social determinant of health that operates as an opportunity structure and provides access to health promoting resources [4]. The neighborhood food environment (e.g., grocery stores), physical activity environment (e.g., parks), and health care environment (e.g., access to health-related services and care) have been shown to be important for health in multiple surveys and epidemiologic cohort studies [5–10]. During the COVID-19 pandemic, these neighborhood resources became especially important for human health and well-being, but they were not equally distributed [11].

As the COVID-19 pandemic took hold, 33 million workers applied for unemployment insurance amid ‘stay at home’ orders designed to safeguard the public and prevent further spread [12]. Yet, 55 million workers continued to provide “essential” services critical to public health and safety, as well as economic and national security [13]. The Department of Homeland Security’s Cybersecurity and Infrastructure Security Agency (CISA) released a list of essential workers in March 2020, defined as those “who conduct a range of operations and services that are essential to continued critical infrastructure viability” [14]. These workers included grocery store clerks, food service workers, hospital custodial staff, construction laborers, home health aides, and other occupations, also known as “frontline” workers, who could not feasibly work from home [15].

Because essential workers were required to continue in-person work, often in high contact, high risk settings, they faced elevated risks [5,16,17]. Indeed, prior work has shown that essential workers experienced higher rates of COVID-19 infection, hospitalization, and death compared to their non-essential worker counterparts [5,6,18]. Frontline workers and their families faced disproportionate COVID exposure risks while also having limited access to personal protective equipment (PPE) and social distancing measures [19], especially early on in the pandemic [20]. Nonetheless, CISA guidance issued on March 19, 2020 noted: “If you work in a critical infrastructure industry… you have a special responsibility to maintain your normal work schedule”. Yet, after leaving work, essential workers returned home to their residential neighborhoods, which varied substantially with regard to the availability of resources that could safeguard health, well-being, and mitigate risk.

Prior research indicates that those who occupy essential work positions tend to be disproportionately socioeconomically disadvantaged, reflecting long-standing structural inequalities in the labor market. Approximately one quarter of essential workers have low household income, 18% live in households with at least one uninsured individual, and 18% live with someone aged 65 years or older [11]. Women and individuals from underrepresented racial and ethnic groups are overrepresented in many essential occupations. For example, women of color hold 60% of housekeeping jobs, 50% of nursing assistant positions, and 46% of personal care aide roles—positions that are often public-facing and involve close contact with others [8,12]. These same structural inequities are closely tied to neighborhood conditions, raising concerns that essential workers may have faced a ‘double burden of vulnerability’: exposure to high-risk work environments while returning to communities with fewer health-promoting amenities during a period when such resources were vital.

Despite extensive research documenting the occupational risks, health outcomes, and characteristics of essential workers during the COVID-19 pandemic [17,19,21], little is known about the neighborhood environments in which essential workers resided, leaving a critical gap in our understanding of their overall risk profile that extends beyond the workplace to include the residential neighborhood context. Specifically, it is not known how access to important neighborhood resources, such as food outlets, health care services, and green space, and other amenities were patterned across neighborhoods with varying proportions of essential workers during the pandemic.

Based on the above, the purpose of this study was to describe, for the first time, the characteristics of US neighborhoods where essential workers were living at the start of the 2020 pandemic and to compare these neighborhoods with those characterized by lower proportions of essential workers. We hypothesized that neighborhoods with a higher concentration of essential workers would demonstrate less favorable access to protective health-promoting resources, such as healthcare services and greenspace, reflecting the broader socioeconomic disadvantage among the essential workforce. We focused on key neighborhood resources critical for health and well-being during the pandemic, including access to food (e.g., grocery stores), health care, and green space (e.g., parks, for socially distanced physical activity). Understanding the community characteristics in which essential workers were living is critical for supporting and preparing the US essential workforce for future disasters and public health emergencies.

## Methods

### Data

Data were obtained from the National Neighborhood Data Archive (NaNDA), a publicly available repository of data on the neighborhood social, demographic, and physical environment across the entire United States [22]. We used data on the residential concentration of essential workers, based on the American Community Survey (ACS) 5-year estimates (2016−2020), linked with data on the characteristics of those neighborhoods at the start of the COVID-19 pandemic (in 2020). All data were obtained at the 2020 census tract level and linked using 11-digit Federal Information Processing Standards (FIPS) codes. Census tracts have relatively stable spatial boundaries which roughly map to neighborhoods. They have a population size between 1,200 and 8,000 people, with an average size of 4,000 people.

### Measures

#### Neighborhood concentration of essential workers.

NaNDA’s Essential Workers dataset [22] is based on 14 occupation categories identified by the American Civil Liberties Union (ACLU) in April 2020 [23] that represent work in the 16 CISA-essential industry sectors [14,24]. This includes workers in healthcare, personal care, and service occupations; transportation and logistics; building maintenance; construction; food preparation and service; agriculture; and protective service occupations. The Essential Workers dataset used census tract data on the employed civilian population (age 16+) by occupation from the ACS five-year estimates (2016–2020) [25,26] and quantified the number of essential workers residing in US census tracts based on ACS Occupation Codes for these 14 occupation categories (see S1 Table). These ACS five year estimates reflect average occupational composition over the 2016–2020 period and therefore do not perfectly capture the occupational composition of neighborhoods at the specific onset of the pandemic in March 2020. However, five year ACS estimates are the smallest geography and most reliable occupational data available at the census tract level. Since neighborhood occupational composition tends to be relatively stable over short time horizons, this measure likely provides a reasonable approximation of essential-worker residential concentration at the pandemic’s onset.

This dataset provides both total counts and proportions of all workers employed in the 14 essential occupations by census tract; for this analysis we used only the proportion variable. The dataset includes a total of 84,373 census tracts (at 2020 census geography) with non-zero employed population (age 16+) in the 50 United States (including Alaska and Hawaii) and Puerto Rico.

#### Neighborhood resource characteristics.

Neighborhood food access was based on a count of the total number of food stores (excluding convenience stores), including supermarkets and grocery stores; meat, fish, and seafood markets; produce markets; and baked goods stores) [27] in each census tract in 2020. We also included data on the number of dollar stores [28] in the census tract, since they were also essential businesses that sold food during the pandemic onset [29]. Access to health care was based on the number of health care offices or clinics in each census tract in 2020. We included data on the total number of physicians’ offices or clinics in each census tract, as well as the number of mental health clinics (offices of mental health physicians, e.g., psychiatrists and psychotherapists; and practitioners, e.g., non-physician clinical psychologists and mental health social workers) [30]. All measures were operationalized as per capita density (count per 1000 population) for analysis. Access to green space was captured using data on the proportion of park area in each census tract in 2020 [31]. This includes publicly owned local, state, and national parks; certain school parks; and privately owned parks that are publicly accessible. The proportion of park area in the census tract is a ratio of the total park area (in square meters) to the total tract area (in square meters). All neighborhood resource characteristics are summarized in S2 Table.

### Analysis

The census tract proportion of essential workers was categorized into quintiles for analysis. Descriptive statistics (means, standard deviations (SD)) were used to summarize each neighborhood characteristic across quintiles of neighborhood essential workers. Heatmaps were used to visualize the distribution across quintiles. Differences in the availability of neighborhood resources between census tracts with the highest (Q5) and lowest (Q1) quintiles of essential workers were evaluated using Welch’s two-sample t-test. This test was chosen because Levene’s test indicated non-homogeneity of variances and non-normal distributions between these groups. Adjacent-quintile pairs were also compared using Welch’s t-test, and a Mann-Kendall monotonic trend test was conducted across all five quintiles for each measure to evaluate whether gradients were consistent across the full distribution (S3 Table). As a sensitivity analysis, comparisons were repeated within metropolitan census tracts only (RUCA4 = 1, based on NaNDA’s Rural-Urban Commuting Area classification) to assess whether observed disparities were driven by urbanicity. Statistical significance was set at α = 0.05. Differences in results were visualized by plotting means and standard error (SE) bars. All analyses were conducted using R (v 4.4.1) within Rstudio [32–34]. Census data were obtained via the tidycensus package (v 1.6.7); data management and visualization used the tidyverse suite (dplyr, tidyr, ggplot2, readr, forcats, purrr).

## Results

Fig 1 summarizes the proportion of essential workers in US census tracts across each national quintile. Boxplots (mean, interquartile range) illustrate that neighborhoods in the lowest quintile had on average 45% (mean = 0.45, SD = 0.086, range = 0–0.56) of the employed population in essential occupations in 2020. In contrast, neighborhoods in the highest quintile had an average of 86% (mean = 0.86, SD = 0.041, range = 0.81–1.0) of the employed population in essential occupations.

**Fig 1:**
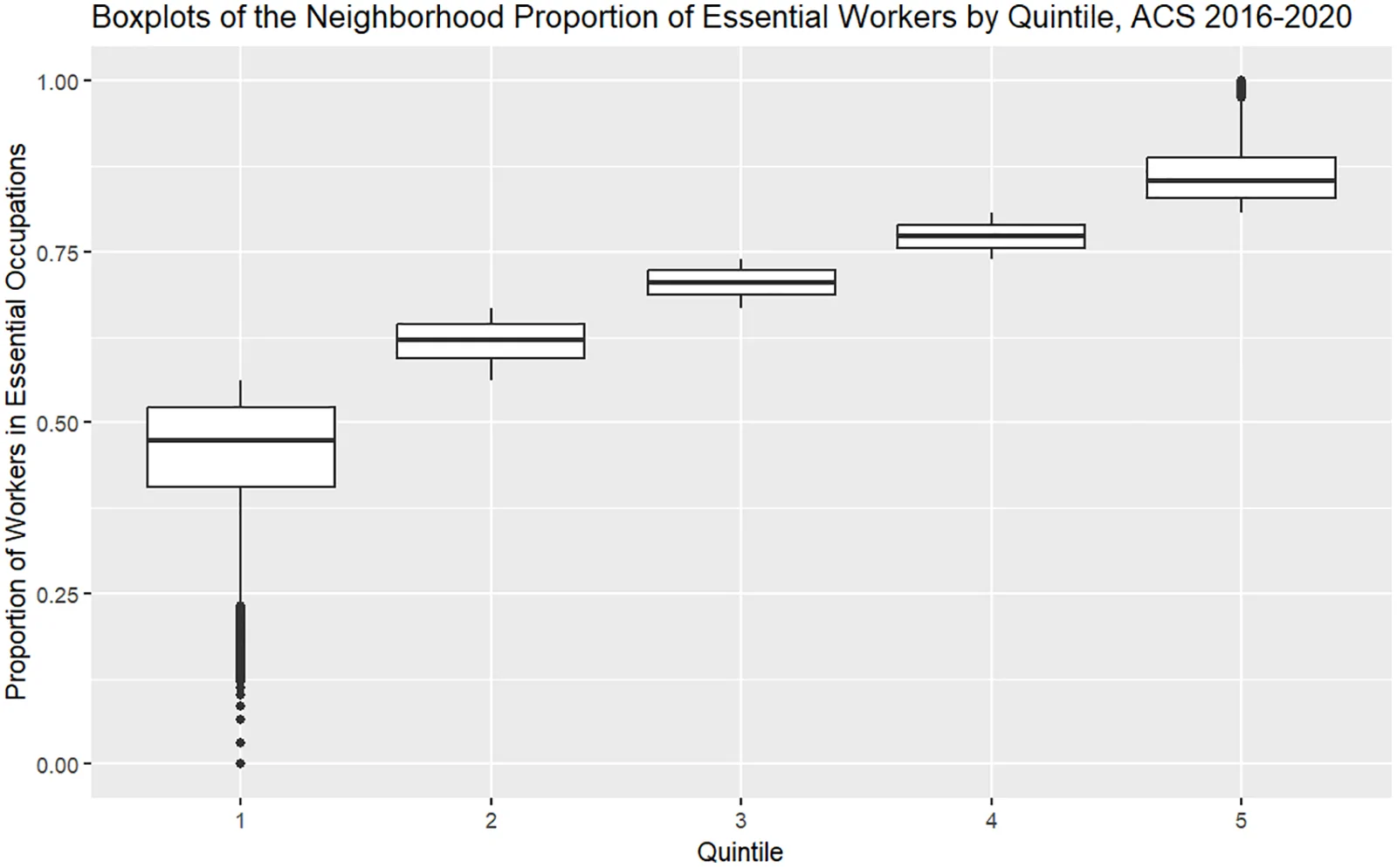
Distribution of essential workers (quintiles) in census tract neighborhoods (ACS 2016-2020).

Table 1 describes the food, healthcare, and green space environments for the neighborhoods where essential workers were living at the start of the 2020 pandemic. Census tract means and standard deviations (SD) are presented for the nation (overall) and by quintiles of essential workers. Compared with census tracts in the lowest essential worker quintile (Q1), those in the highest quintile (Q5) had lower median family incomes (mean = $50,493 vs. $134,939), higher poverty rates (23.7% vs. 7.6%), lower bachelor’s degree attainment (13.0% vs. 60.2%), and a more racially and ethnically diverse population, with lower proportions of non-Hispanic White residents (41.0% vs. 68.1%) and higher proportions of non-Hispanic Black (21.6% vs. 7.3%) and Hispanic residents (31.2% vs. 10.4%). On average, there were 0.43 (SD = 3.67) food stores per 1000 population in these census tract neighborhoods. The density of food stores was highest in neighborhoods with the largest proportion of essential workers (Q5) with an average of 0.59 food stores per 1000 population, and lowest in neighborhoods in the bottom second and third quintiles (with an average of 0.35 and 0.36 food stores per 1000 population, respectively). Neighborhood dollar stores were less common than food stores (0.10 (SD = 0.30) per 1000 pop overall), but in neighborhoods with higher proportions of essential workers dollar stores were more plentiful than in neighborhoods with fewer essential workers (e.g., 0.16 vs 0.03 dollar stores per 1000 population in Q5 vs Q1, respectively).

**Table 1:** Residential neighborhood characteristics for the United States essential workforce: 2020 (N = 84,373 Census Tracts).

		Census Tract Proportion of Workers in Essential Occupations (National Quintiles)				
	Overall Mean (SD)	Q1 Mean (SD)	Q2 Mean (SD)	Q3 Mean (SD)	Q4 Mean (SD)	Q5 Mean (SD)
**Demographic Characteristics**						
Median Family Income ($)	$83,514 ($40,695)	$134,939 ($46,169)	$92,808 ($27,766)	$75,502 ($21,894)	$63,893 ($18,840)	$50,493 ($17,265)
Poverty Rate	0.142 (0.120)	0.076 (0.082)	0.101 (0.095)	0.129 (0.099)	0.165 (0.110)	0.237 (0.135)
Bachelor’s Degree or Higher	0.318 (0.197)	0.602 (0.149)	0.385 (0.116)	0.271 (0.097)	0.201 (0.082)	0.130 (0.072)
Non-Hispanic White	0.603	0.681	0.677	0.651	0.595	0.410
Non-Hispanic Black	0.129	0.073	0.095	0.115	0.145	0.216
Hispanic	0.178	0.104	0.134	0.155	0.186	0.312
Food Environment*						
Food Stores	0.426 (3.67)	0.421 (2.02)	0.347 (0.53)	0.365 (0.48)	0.405 (1.06)	0.594 (7.85)
Dollar Stores	0.102 (0.30)	0.028 (0.10)	0.068 (0.16)	0.108 (0.20)	0.142 (0.22)	0.164 (0.56)
Healthcare Environment*						
Physicians’ Offices	1.154 (12.09)	1.84 (7.23)	1.35 (23.8)	0.91 (2.20)	0.81 (2.36)	0.91 (9.99)
Mental Health Physicians’ Offices	0.023 (0.28)	0.053 (0.24)	0.027 (0.56)	0.014 (0.87)	0.010 (0.77)	0.009 (0.08)
Mental Health Practitioners’ Offices	0.097 (0.65)	0.252 (1.18)	0.102 (0.66)	0.056 (0.22)	0.039 (0.16)	0.034 (0.43)
**Green Space (N = 83451)**						
Proportion of Tract Area that is Park land	0.043 (0.10)	0.063 (0.11)	0.049 (0.11)	0.038 (0.09)	0.033 (0.09)	0.031 (0.08)

* Expressed as the number per 1000 population in the census tract

In contrast, the availability of health care was less common in neighborhoods with a greater proportion of essential workers (Table 1). While the overall density of physicians’ offices was 1.15 (SD = 12.09) per 1000 population in census tracts nationwide, the number of physicians’ offices was highest in neighborhoods with the fewest essential workers (1.84 per 1000 population in Q1) and was lower in neighborhoods with a higher proportion of the essential workforce (0.91 per 1000 population in Q5). A similar pattern was observed for mental health physicians (psychiatrists and psychotherapists), although the density of these resources in census tract neighborhoods was relatively small overall (0.02 (SD = 0.28) per 1000 population overall). Offices of mental health practitioners were more common in US neighborhoods overall (0.10 (SD = 0.65), Table 1) but tended to be more heavily located in census tracts with a lower proportion of essential workers (0.25 per 1000 population in Q1) compared to neighborhoods with the highest proportion of essential workers (vs 0.03 per 1000 population in Q5). Green space was also patterned differently across essential worker neighborhoods. In neighborhoods with the highest proportion of essential workers, parks comprised an average of 3% of the total area of the census tract (Q5 mean (SD) = 0.031 (0.08)) compared to an average of 6% of the total land area of census tracts in which the fewest proportion of essential workers were living (Q1 mean (SD) = 0.063 (0.11), Table 1).

Consistent with the pairwise comparisons above, Mann-Kendall trend tests confirmed monotonic gradients across all five quintiles and neighborhood resources. Food stores (τ = 0.070) and dollar stores (τ = 0.194) increased significantly with essential worker concentration, while physicians’ offices (τ = −0.131), mental health physicians’ offices (τ = −0.118), mental health practitioners’ offices (τ = −0.194), and park land (τ = −0.120) all decreased significantly, indicating that these disparities were not confined to the extreme quintiles but reflected a consistent gradient across the full distribution (See S3 Table).

Fig 2 further illustrates these differences in a heatmap showing the variability in resources (standardized mean) available across neighborhoods with different proportions of essential workers. Neighborhoods with the highest proportion of essential workers (Q5) had the highest density of food stores. Neighborhoods with the lowest proportion of essential workers (Q1) had the highest density of health care resources, including offices/clinics of mental health physicians and practitioners, and had the greatest proportion of park area in their census tract neighborhoods.

**Fig 2:**
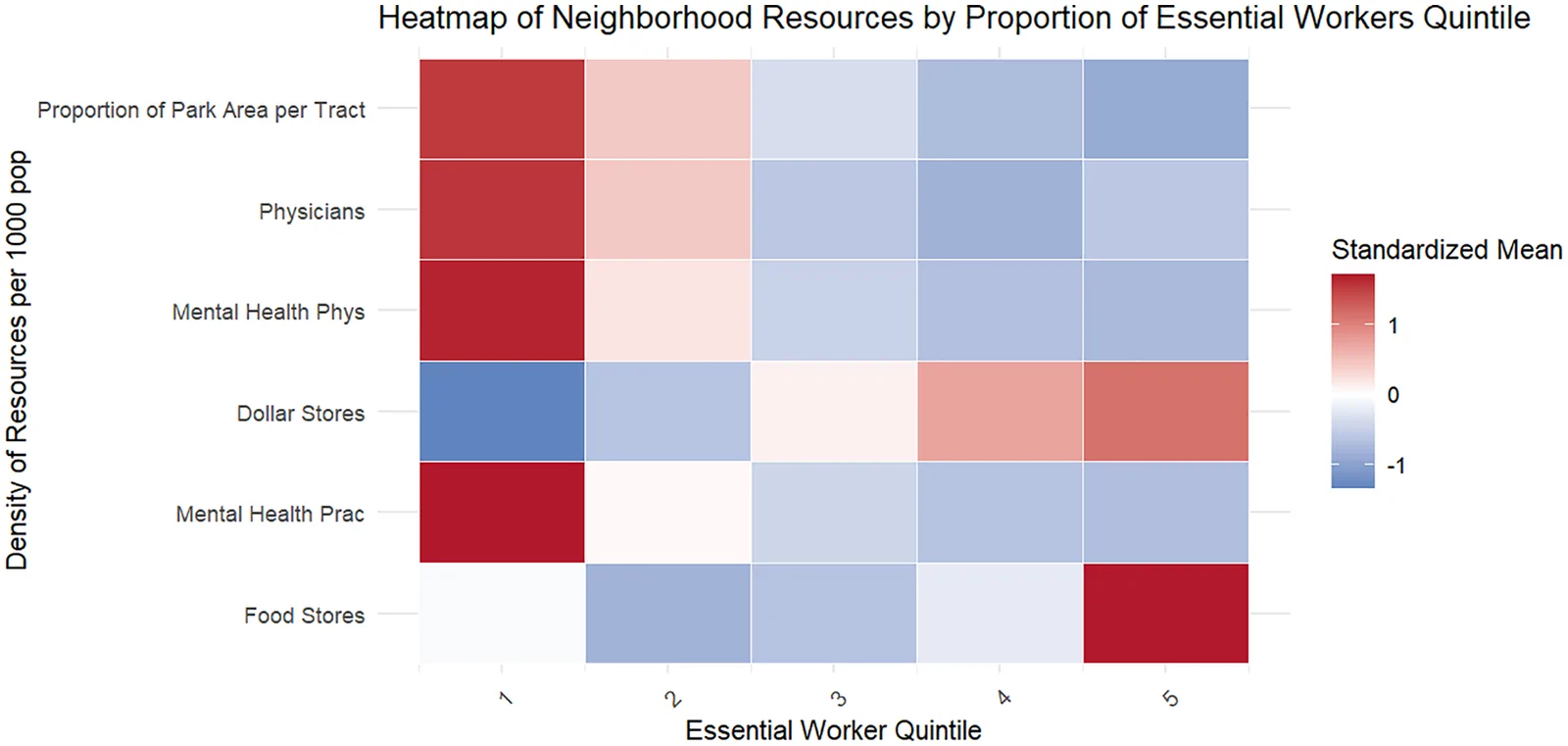
Heatmap of neighborhood resources by essential worker quintile (2020).

Figs 3–5 present the differences in means (with standard errors bars) of resources available in census tracts with the highest (Q5) and lowest (Q1) proportion of essential workers in 2020. Fig 3 illustrates the results from the t-test comparing means of the density of food stores. Neighborhoods with the highest proportion of essential workers (Q5) had significantly higher density of food stores (mean = 0.59 per 1000 population, SE = 0.06) than neighborhoods with the lowest proportion of essential workers (mean = 0.42, SE = 0.02; t = 2.77, p < 0.01). They also had significantly more dollar stores (mean (SE) = 0.16 (0.004)) compared to neighborhoods with the lowest proportion of essential workers (mean (SE) = 0.03 (0.001), t = 31.07, p < 0.001).

**Fig 3:**
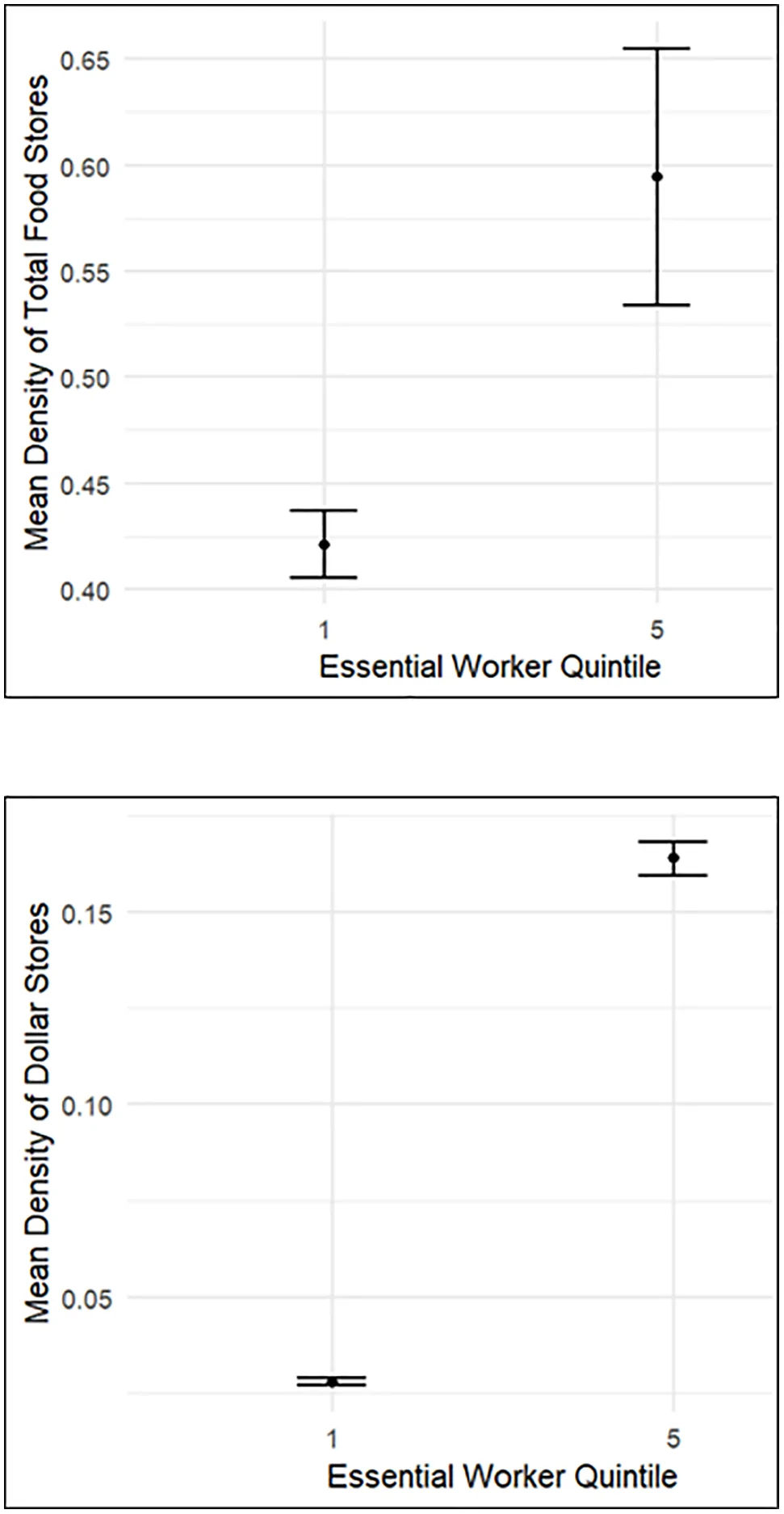
Density (per 1000 population) of food stores in neighborhoods with the highest (Q5) and lowest (Q1) proportion of essential workers (2020): Means and standard error bars.

**Fig 4:**
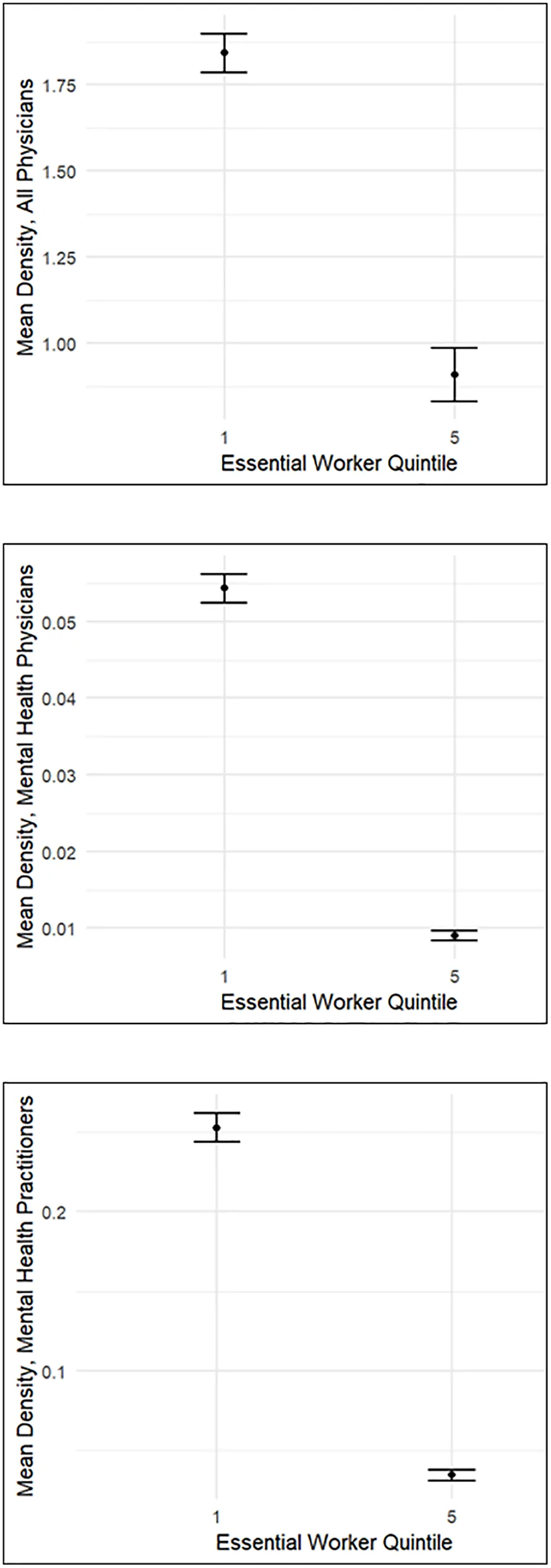
Density (per 1000 population) of health care resources in neighborhoods with the highest (Q5) and lowest (Q1) proportion of essential workers (2020): Means and standard error bars.

**Fig 5:**
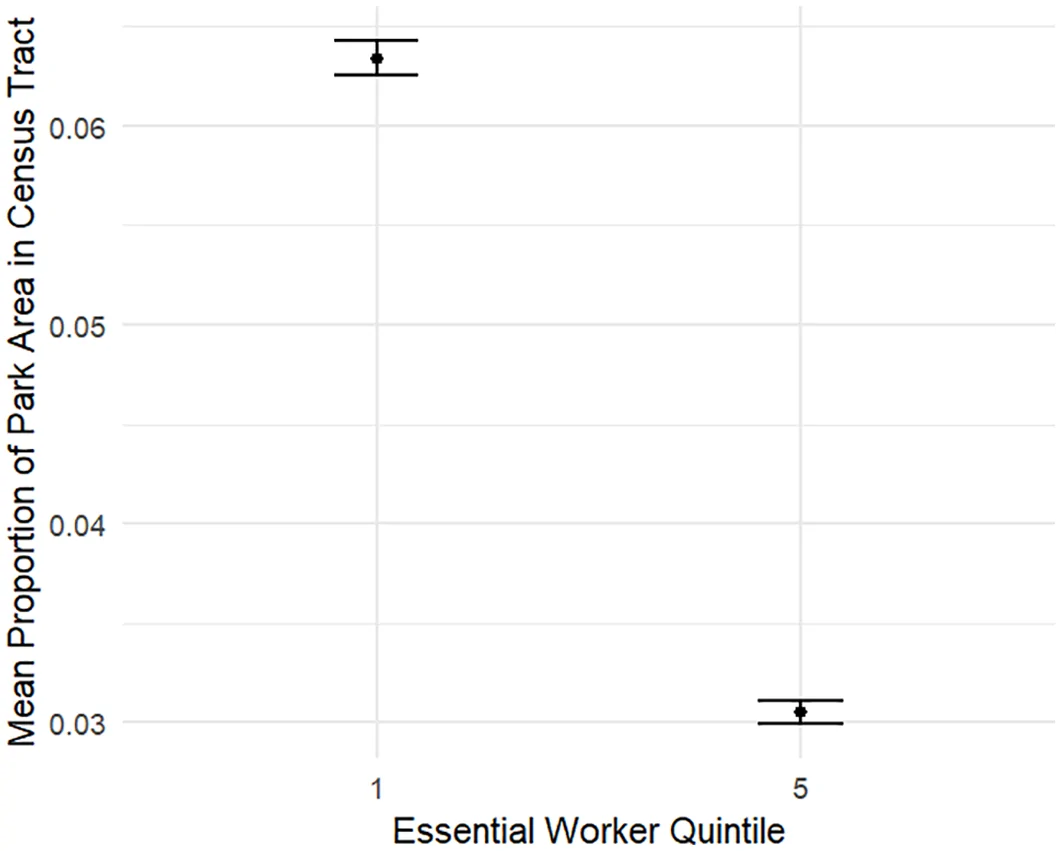
Mean proportion of park area in census tracts with the highest (Q5) and lowest (Q1) proportion of essential workers (2020): Means and standard error bars.

Fig 4 presents the difference in health care resources available in census tracts with the highest and lowest proportion of essential workers. Compared to neighborhoods with the lowest proportion of essential workers (Q1), neighborhoods with the greatest concentration of essential workers (Q5) had significantly fewer physicians’ offices (mean (SE) = 0.91 (0.08) vs. 1.84 (0.06) per 1000 population; t = 9.82, p < 0.001). The availability of mental health care resources was also significantly lower in neighborhoods with a high proportion of essential workers. On average, there were 0.01 mental health physicians and 0.03 mental health practitioners per 1000 population in neighborhoods with the highest proportion of essential workers (Q5), compared to 0.05 and 0.25 per 1000, respectively, in neighborhoods with fewer essential workers (t > 22.5 for both comparisons, p < 0.001).

The difference in the proportion of park area between the highest and lowest quintiles of essential worker neighborhoods is illustrated in Fig 5. In neighborhoods with the highest proportion of essential workers (Q5), an average of 3% (mean (SE) = 0.03 (0.001)) of the census tract area was park land. But in neighborhoods with the lowest proportion of essential workers (Q1), the average proportion of the census tract devoted to park land was double that (mean (SE) = 0.06 (0.001); t = 30.86, p < 0.001).

To assess whether these findings were driven by differences in urbanicity, we conducted a sensitivity analysis restricted to metropolitan census tracts (RUCA4 = 1), which produced results that were consistent with the primary analysis. Compared with tracts in the lowest essential worker quintile (Q1), metropolitan tracts in the highest quintile (Q5) continued to have lower densities of physicians’ offices, mental health physicians’ offices, and mental health practitioners’ offices, as well as lower proportions of park land, but higher densities of total food stores and dollar stores (all p < 0.01). These findings suggest that the observed neighborhood resource disparities were not solely attributable to differences in urbanicity.

## Discussion

This is the first empirical examination of the neighborhood characteristics in which the US essential workforce was living at the start of the COVID-19 pandemic. While previous research has documented the characteristics of essential workers themselves, understanding the environment that these workers returned home to each day is necessary for fully understanding the challenges and resources faced by this important part of the US pandemic response. Using nationwide data on the census tract characteristics in which essential workers lived in 2020, we found that access to food stores was generally higher in neighborhoods with a high proportion of essential workers, but access to health care and parks/greenspace was significantly lower than in neighborhoods characterized by fewer essential workers.

The scale and severity of disasters in the US are growing, and workers in frontline or “essential” occupations will continue to be called upon to work in the face of systemic environmental or public health threats [35]. In 2011 the US Federal Emergency Management Agency (FEMA) shifted to a ‘Whole Community Approach’ to disaster management in recognition that a government-centric approach was not sufficient to meet the needs of all communities [35]. Access to food, healthcare, and greenspace are important for building resilient communities in the face of a disaster or emergency. By identifying the community resources that were and were not available to support essential workers during the COVID-19 pandemic, this paper can inform future disaster planning and preparedness.

Groceries were in short supply at the beginning of the COVID-19 pandemic, and access to healthy foods, such as fruits and vegetables, were especially scarce as consumers shifted to in-home meal preparation while restaurants closed. Communities that already had higher rates of food insecurity before the pandemic were disproportionally affected, including households with children, single mother households, and non-Hispanic Black households [36]. We found that essential worker neighborhoods were fortunate to have better access to health food stores than neighborhoods with fewer essential workers. However, we were unable to identify differences in food store availability among essential workers from different social and demographic groups. Dollar stores played a key role in the pandemic by selling food, particularly in rural, low-income, and under-served urban communities where traditional grocery stores were in decline well before the pandemic onset [37]. We found that essential worker neighborhoods not only had more grocery stores but also dollar stores as well, supporting their communities to withstand and recover from the pandemic disruption.

While essential worker neighborhoods were more likely to have greater access to food, they tended to have lower access to health care and green space relative to neighborhoods with fewer essential workers. Access to healthcare was complicated during the pandemic onset, when doctors’ offices closed and the use of healthcare services declined [38]. However, the fact that neighborhoods with a higher concentration of essential workers had fewer health care resources raises questions about the ability of those workers to maintain good health and access primary care prevention in advance of the pandemic. Maintaining health, including screenings and preventive services, is necessary for supporting the essential workforce as part of a whole community emergency preparedness. We found that the distribution of mental health services was notably weaker across communities with a greater proportion of essential workers during the pandemic. The COVID-19 pandemic witnessed a surge in mental health issues, particularly depression and anxiety, as lockdowns and social distancing took their toll [39]. Yet, the nation’s essential workforce, who was at higher risk for mental distress [40], did not have the same availability of mental health services in their communities, particularly mental health practitioners, as communities with fewer essential workers.

With the onset of the COVID-19 pandemic, many neighborhood physical activity resources became off-limits to residents as gyms and community centers closed or operated with tight restrictions. But neighborhood parks and green space continued to promote opportunities for physically distanced activity, social interaction, and well-being. Yet, we found that the neighborhoods where essential workers lived were less likely to have park area than neighborhoods with fewer essential workers.

Supporting the essential workforce not only entails the provision of food and healthcare services, but also the opportunities for physically distanced social interaction, recreational walking, and the mental health benefits of green space [41]. Additionally, disparities observed in this study likely reflect both urbanicity and socioeconomic disadvantage: essential worker concentration was associated with more urban tracts and substantially lower median household income (Q1: $134,939 vs. Q5: $50,493), though healthcare and greenspace gaps persisted even within metropolitan areas alone. We also caution that greater food-store density, driven largely by dollar stores, does not necessarily reflect healthier or more affordable food access.

Previous research has identified individual level policy implications relevant for the essential workforce during the COVID-19 pandemic: “Policies to support workers who are putting themselves and their families at risk to benefit others should be considered, and there is urgent need for economic analyses of the costs and benefits of approaches such as stimulus payments, short-term health care coverage, and prioritized testing” [42]. However, addressing the context in which essential workers lived was generally ignored. Preparing for the support of the essential workforce during the next pandemic or disaster must also include a consideration of their neighborhood context.

### Strengths and limitations

The novel national scope and tract-level granularity of this nationwide linkage of neighborhood essential worker density with neighborhood healthcare, food and greenspace characteristics at the start of the COVID-19 pandemic offers new insights into structural inequalities that shape occupation exposure, not only during pandemics, but as a feature of American labor geography. It additionally provides a lens into the spatial and social patterns of occupation-related risk and offers insights into how neighborhood-level factors may exacerbate disparities in disease exposures and resilience.

Despite these strengths, this study has some limitations that should be noted. First, it is unknown whether essential workers actually used the resources in their neighborhoods. Examining where essential workers reside relative to their place of employment (or resources in employment neighborhoods) is the subject of future research. Relatedly, our measures reflect the supply, or density, of neighborhood resources rather than demand or adequacy relative to the local population. In other words, a quintile with comparatively lower resource density may still be sufficient to meet residents’ needs, while a quintile with higher density may remain undersupplied relative to demand. Our measures also capture quantity but not quality, affordability, or accessibility. For example, a greater density of food retail outlets does not indicate whether residents can access affordable, healthy food, and a greater density of physicians’ offices does not indicate whether those practices accept new patients or relevant insurance types.

Second, the variability observed within some essential worker quintiles reflects both substantive neighborhood heterogeneity and a known property of per-capita density measures at the census tract level: tracts with a small residential population but a concentrated institutional presence (i.e., a hospital campus or medical office cluster) can generate extreme per capita outliers even within an otherwise consistent quintile. Average differences between quintiles should therefore be interpreted alongside this within group variation; access to neighborhood resources is not uniformly distributed even among tracts with similar essential worker concentration.

Third, this study did not examine several additional neighborhood domains relevant to well-being, including, but not limited to, public transportation, faith based and community/recreational spaces, as these data were not available for this analysis, nor did our health care measure include hospitals, emergency departments, urgent care, dental, or physical/occupational therapy services. However, we note that many of these resources were broadly curtailed nationwide by pandemic era restrictions regardless of local density.

Lastly, this study was a neighborhood level analysis that was both exploratory and descriptive. As a result, we did not examine health outcomes among essential workers as a function of neighborhood resource access. Future research should test whether these access differences translate into meaningful differences in health behaviors and should incorporate measures of resource affordability and quality in order to more fully understand the opportunities and barriers for improving essential worker community health.

## Conclusions

Preparing for the support of the essential workforce during the next pandemic, or disaster must also include consideration of their neighborhood context. Our findings may inform the development of community-level risk management tools, which will help build a more resilient essential worker community in the face of future disasters and public health emergencies.

## Supporting information

S1 Table American community survey (ACS) occupation codes for essential workers in NaNDA’s Essential Worker Dataset (ACS 2016–2020). Essential occupational categories, as defined by the American Civil Liberties Union (2020), and their corresponding ACS occupation codes.
(DOCX)

S2 Table Neighborhood resource characteristics. Definitions and data sources for the neighborhood food access, health care access, and green space measures used in this study.
(DOCX)

S3 Table Mann-Kendall tests for trends in neighborhood characteristics across essential workforce quintiles. Results of Mann-Kendall monotonic trend tests evaluating gradients in neighborhood resources across all five quintiles of essential worker concentration.
(DOCX)
