# Supplementary material for: Essential work, unequal places: Neighborhood characteristics of the essential workforce in the United States during the COVID-19 pandemic
Source: PLoS One. 2026 Sep 30;21(9):e0357792. doi: 10.1371/journal.pone.0357792 (PMC13626339; doi:10.1371/journal.pone.0357792)
Supplement: Supplementary file 1 [file pone.0357792.s001.docx]

**S1 Table. American Community Survey (ACS) Occupation Codes for Essential Workers in NaNDA’s Essential Worker Dataset (ACS 2016-2020).**

| **Essential Occupational Category** | **ACS Occupation Codes** | **ACS Occupation Codes** |
| --- | --- | --- |
| Healthcare practitioners and technical occupations | c24010e16 | c24010e52 |
| Construction and extraction occupations | c24010e32 | c24010e68 |
| Farming, fishing, and forestry occupations | c24010e31 | c24010e67 |
| Installation, maintenance, and repair occupations | c24010e33 | c24010e69 |
| Material moving occupations | c24010e37 | c24010e73 |
| Production occupations | c24010e35 | c24010e71 |
| Transportation occupations | c24010e36 | c24010e72 |
| Office and administrative support occupations | c24010e29 | c24010e65 |
| Sales and related occupations | c24010e28 | c24010e64 |
| Building and grounds cleaning and maintenance occupation | c24010e25 | c24010e61 |
| Food preparation and serving related occupations | c24010e24 | c24010e60 |
| Healthcare support occupations | c24010e20 | c24010e56 |
| Personal care and service occupations | c24010e26 | c24010e62 |
| Protective service occupations | c24010e21 | c24010e57 |

*As defined by the American Civil Liberties Union (2020).*
