# Supplementary material for: Essential work, unequal places: Neighborhood characteristics of the essential workforce in the United States during the COVID-19 pandemic
Source: PLoS One. 2026 Sep 30;21(9):e0357792. doi: 10.1371/journal.pone.0357792 (PMC13626339; doi:10.1371/journal.pone.0357792)
Supplement: Supplementary file 2 [file pone.0357792.s002.docx]

**S2 Table. Neighborhood Resource Characteristics**

Neighborhood characteristics included food access (Table 2a), health care access (Table 2b), and green space (Table 2c). All count-based measures were standardized as counts per 1,000 population; park land was expressed as a proportion of total census tract area.

**Table 2a. Food Access Measures**

| **Measure** | **Components** | **Source** |
| --- | --- | --- |
| Food stores | Supermarkets, grocery stores, meat/fish/seafood markets, produce markets, bakeries (excluding convenience stores) | Melendez et al., 2024b |
| Dollar stores | Dollar stores | Melendez et al., 2024a |

**Table 2b. Health Care Access Measures**

| **Measure** | **Components** | **Source** |
| --- | --- | --- |
| Physicians' offices/clinics | All physicians' offices and clinics | Melendez et al., 2024 |
| Mental health clinics | Offices of psychiatrists, psychotherapists, clinical psychologists, mental health social workers | Melendez et al., 2024 |

**Table 2c. Green Space Measures**

| **Measure** | **Components** | **Source** |
| --- | --- | --- |
| Park area | Public parks (local, state, national), accessible school parks, publicly accessible private parks | Melendez et al., 2022 |
