# Supplementary material for: Essential work, unequal places: Neighborhood characteristics of the essential workforce in the United States during the COVID-19 pandemic
Source: PLoS One. 2026 Sep 30;21(9):e0357792. doi: 10.1371/journal.pone.0357792 (PMC13626339; doi:10.1371/journal.pone.0357792)
Supplement: Supplementary file 3 [file pone.0357792.s003.docx]

**S3 Table. Mann-Kendall Tests for Trends in Neighborhood Characteristics Across Essential Workforce Quintiles.**

|  | **Kendall's τ** | **Z** | **S** | **p-value** | **Trend Direction** |
| --- | --- | --- | --- | --- | --- |
| **Food Environment*** |  |  |  |  |  |
| Food Stores | 0.070 | 29.111 | 2.314x10^8^ | p < 2.2x10^-16^ | Increasing |
| Dollar Stores | 0.194 | 74.056 | 4.766x10^8^ | p < 2.2x10^-16^ | Increasing |
| **Healthcare Environment*** |  |  |  |  |  |
| Physicians’ Offices | -0.131 | -54.256 | -4.294x10^8^ | p < 2.2x10^-16^ | Decreasing |
| Mental Health Physicians’ Offices | -0.118 | -42.479 | -1.412x10^8^ | p < 2.2x10^-16^ | Decreasing |
| Mental Health Practitioners’ Offices | -0.194 | -71.97 | -3.765x10^8^ | p < 2.2x10^-16^ | Decreasing |
| **Green Space (N = 83451)** |  |  |  |  |  |
| Proportion of Tract Area that is Park land | -0.120 | -50.161 | -3.958x10^8^ | p < 2.2x10^-16^ | Decreasing |

** Density measures expressed as the number per 1000 population in the census tract.*
